# Left atrial structure and functional quantitation using cardiac magnetic resonance: comparison of manual delineation vs. multimodality tissue tracking based semi-automated methods

**DOI:** 10.1186/1532-429X-16-S1-P348

**Published:** 2014-01-16

**Authors:** Mytra Zareian, Mohammadali Habibi, Bharath Ambale Venkatesh, Anders Opdahl, Elzbieta H Chamera, Colin Wu, Filip Zemrak, David Bluemke, Joao A Lima

**Affiliations:** 1 Cardiovascular Imaging, Johns Hopkins University, Baltimore, Maryland, USA; 2 Cardiology, University of Oslo, Oslo, Norway; 3 National Institutes of Health, Bethesda, Maryland, USA; 4 National Institutes of Health, London, UK

## Background

Left atrium (LA) volume and function are important markers of cardiovascular disease. LA volume can be assessed by several different methods. In clinical practice, the Simpson's method is well accepted as a reference standard, although there is no standardization for LA volume calculations. We aimed to compare the estimations of LA volume by the Simpson's method and the modified biplane Simpson's method; and to introduce Multimodality Tissue Tracking (MTT, Japan, Toshiba) as a new semi-automated method for quantifying LA function based on tissue feature tracking.

## Methods

**Figure 1:**
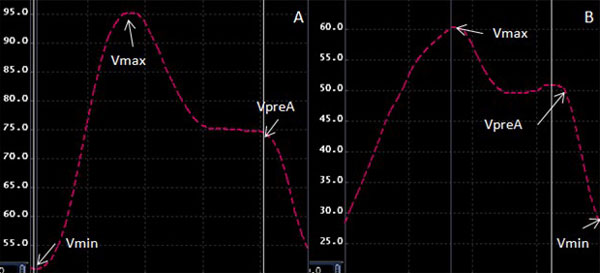
**Multimodality Tissue Tracking, volume analysis**. Subject with heart failure (Panel A) versus a healthy subject (Panel B).Vmax; maximal volume, VpreA; volume before pre-atrial contraction, Vmin; minimal volume.

Thirty subjects (mean age: 71.3 ± 8.7, 87% male) including twenty subjects with cardiovascular events (4 atrial fibrillation, 18 myocardial scar from late gadolinium enhancement, 2 heart failure) and ten healthy subjects, with CMR imaging were evaluated in the Multi-Ethnic Study of Atherosclerosis (MESA). LA volumes were measured using the modified biplane Simpson's method from 2- and 4-chamber projections and the original Simpson's method using short-axis slices. For the manual methods, LA endo- and epicardial boundaries were delineated at left-ventricular end-diastole (Vmin), end-systole (Vmax) and just before the pre-atrial contraction (VpreA). Using MTT, LA endocardial and epicardial borders were manually delineated at end-systole and the boundaries were propagated automatically throughout the cardiac cycle. LA total (LAEF = Vmax-Vmin/Vmax), active (LAAEF = VpreA-Vmin/VpreA) and passive ejection fraction (LAPEF =Vmax-VpreA/Vmax) were calculated (Figure 1). A two-tailed paired sample t-test is used to determine significant differences between two types of computations (Simpson's from short-axis images vs. Biplane Simpsons from long-axis images) and methods (MTT vs. manual). Pearson's correlation and Bland-Altman analysis are used to examine the relationship between the two computations and methods. In addition, intra-class correlation coefficients (ICC) for inter and intra reader reproducibility are calculated.

## Results

LA parameter analysis using the two different computations was significantly different for functional LA parameters by both methods (Table 1). LA functional parameters did not differ between manual and semi-automated methods (Table 1). LA volumes obtained from the Simpson's method was significantly different between manual and MTT methods (Table 1). Image analysis was less time consuming on average with MTT (Simpson's: MTT vs. manual: 3:10 min vs. 7:23 min; Biplane, MTT vs. manual: 1:30 min vs. 8:28 min). All parameters showed good to excellent intra and inter reader reproducibility (ICC; 0.69-0.99).

**Table 1:** Comparison between the Simpson's method vs. Biplane method, Manual vs. MTT

	Biplane	Simpson's	p	r	p
Manual					

Vmax (ml)	84.3 ± 34.6	88.1 ± 35.2	0.08	0.95	< 0.001

LAEF (%)	0.44 ± 0.1	0.34 ± 0.1	< 0.001	0.88	< 0.001

LAPEF (%)	0.16 ± 0.08	0.11 ± 0.05	< 0.001	0.70	< 0.001

LAAEF(%)	0.37 ± 0.07	0.28 ± 0.07	< 0.001	0.70	< 0.001

MTT					

Vmax (ml)	86.5 ± 33.6	85.2 ± 35.2	0.53	0.95	< 0.001

LAEF (%)	0.46 ± 0.2	0.33 ± 0.10	< 0.001	0.85	< 0.001

LAPEF (%)	0.18 ± 0.05	0.11 ± 0.04	< 0.001	0.57	0.002

LAAEF(%)	0.38 ± 0.08	0.27 ± 0.06	< 0.001	0.61	< 0.001

	MTT	Manual	p	r	p

Biplane					

Vmax (ml)	86.5 ± 33	84.4 ± 34	0.08	0.98	< 0.001

LAEF (%)	0.45 ± 0.13	0.44 ± 0.12	0.21	0.88	< 0.001

LAPEF (%)	0.17 ± 0.05	0.16 ± 0.08	0.67	0.57	0.002

LAAEF(%)	0.16 ± 0.08	0.36 ± 0.09	0.21	0.83	< 0.001

Simpson's					

Vmax (ml)	85.2 ± 35.2	88.2 ± 35.2	< 0.001	1.00	< 0.001

LAEF (%)	0.33 ± 0.1	0.34 ± 0.1	0.19	0.92	< 0.001

LAPEF (%)	0.11 ± 0.04	0.11 ± 0.05	0.43	0.29	0.14

LAAEF(%)	0.27 ± 0.06	0.28 ± 0.06	0.52	0.86	< 0.001

Results are reported as mean ± standard deviation. Abbreviations: Vmax; maximal volume; LAEF, left atrial total ejection fraction; LAPEF, left atrial passive ejection fraction; LAAEF, left atrial active ejection fraction; Smax, maximal global strain (a longitudinal strain vs. b circumferential strain); r, Pearson's correlation.

## Conclusions

MTT derived biplane LA structure and function is accurate, less time consuming, highly reproducible and could potentially be used in large studies.

## Funding

NHLBI N01-HC-95159 NHLBI N01-HC-95168 NCRR UL1-RR-024156 NCRR UL1-RR-025005.

